# Prevalence, risk characteristics, and prediction of low-dose edoxaban treatment in hospitalized patients: a multicenter, observational cohort study

**DOI:** 10.3389/fphar.2025.1427634

**Published:** 2025-04-28

**Authors:** Shujuan Zhao, Hengfen Dai, Jiaxin Chen, Ming Ni, Wenxing Peng, Xiaoyu Li, Fen Li, Boya Chen, Haixia Cai, Yinping Liu, Song Du

**Affiliations:** ^1^ Department of Pharmacy, Henan Provincial People’s Hospital, School of Clinical Medicine, People’s Hospital of Zhengzhou University, Henan University, Zhengzhou, Henan, China; ^2^ Department of Pharmacy, Affiliated Fuzhou First Hospital of Fujian Medical University, Fuzhou, Fujian, China; ^3^ Department of Pharmacy, Ningde Municipal Hospital Affiliated to Ningde Normal University, Ningde, Fujian, China; ^4^ Department of Clinical Pharmacy, Fuwai Central China Cardiovascular Hospital, Zhengzhou, Henan, China; ^5^ Department of Pharmacy, Beijing Anzhen Hospital of Capital Medical University, Beijing, China; ^6^ Department of Pharmacy, Central’s Hospital of Xinxiang, Xinxiang, Henan, China; ^7^ Department of Pharmacy, The First People’s Hospital of Xinxiang, Xinxiang, Henan, China; ^8^ Department of Cardiovascular Medicine, Henan Provincial People’s Hospital, School of Clinical Medicine, People’s Hospital of Zhengzhou University, Henan University, Zhengzhou, Henan, China

**Keywords:** non-vitamin K antagonist oral anticoagulants, low-dose, risk factors, prediction model, LASSO, bootstrap

## Abstract

**Background:**

Treatment with a low-dose non-vitamin K antagonist oral anticoagulant (NOAC) is common among hospitalized patients, and a model to predict the need for such treatment would support individualized interventions. This study evaluated the prevalence of low-dose edoxaban treatment and developed and evaluated a model to predict low-dose administration of edoxaban among hospitalized patients.

**Methods:**

This study included 1208 inpatients with non-valvular atrial fibrillation (NVAF) or venous thromboembolism (VTE) who were treated with edoxaban. Univariate and multivariate analyses identified variables significantly associated with low-dose edoxaban therapy. Least absolute shrinkage and selection operator (LASSO) regression was used for data dimension reduction and selection of the best variables. A nomogram was built based on the predictive variables for easy visualization. Model performance was evaluated, and the model was further validated internally with 1000 bootstrap resamples.

**Results:**

The prevalence of low-dosing edoxaban treatment was 65.98% (797/1208). The predictors of edoxaban included in the final nomogram were age, weight, surgery or operation, anticoagulation indication, the use of antiarrhythmic drugs, anemia, and bleeding history. The model showed good discrimination with an area under the curve value of 0.792. The Hosmer‒Lemeshow test showed that the model had satisfactory goodness of fit (χ^2^ = 10.757, P = 0.2158). The calibration curve showed good agreement between predicted and actual probabilities.

**Conclusion:**

The developed predictive model for low-dose edoxaban use among hospitalized patients was built using seven readily available variables and showed good performance. This study provides an empirical basis for early detection and intervention using a low-dose NOAC.

## 1 Introduction

Non-vitamin K antagonist oral anticoagulants (NOACs), also known as direct oral anticoagulants (DOACs), are applied in clinical practice for several indications, including non-valvular atrial fibrillation (NVAF) and venous thromboembolism (VTE), to reduce the risk of stroke or thromboembolism (Bejjani et al., 2024). As a supplement with vitamin K antagonists (VKAs), NOAC usage has rapidly expanded worldwide during the last decade (Camm et al., 2020).

Currently, four main types of NOACs are available for clinical use: dabigatran, rivaroxaban, apixaban, and edoxaban (Zhao et al., 2020a). Edoxaban is a direct Factor Xa inhibitor with distinct pharmacokinetic properties compared with other NOACs. It achieves peak plasma concentrations within 1–2 h after oral administration and has a half-life of approximately 10–14 h. Edoxaban is primarily eliminated via renal excretion (∼50%) and non-renal pathways. In contrast with other factor Xa inhibitor, the minimal reliance (∼4%) of edoxaban on the cytochrome p450 enzyme system reduces the risk of drug–drug interactions. Moreover, apixaban and dabigatran require twice-daily dosing, whereas the once-daily edoxaban regimen offers potential advantages in terms of patient adherence (Zhao et al., 2020b).

As the most recently approved NOAC of these agents, edoxaban use following NVAF or VTE has been evaluated in historical randomized controlled trials (RCTs) (Büller et al., 2013; Giugliano et al., 2013; Raskob et al., 2018). Generally, an edoxaban dose of 60 mg once daily is indicated, but dose adjustments may be needed according to individual patient characteristics, particularly renal insufficiency, low body weight, or drug–drug interactions (Choi et al., 2024). In this context, the dosage rules for the correct prescription may be to a certain extent poorly understood or intentionally neglected due to clinical heterogeneity and various potential individual features, and this may result in inappropriate administration (underdosing or overdosing) (Zhao et al., 2024).

The majority of dose adjustments of NOACs in clinical practice are inconsistent with guideline-relevant recommendations, and studies have found that under-dosing seems to be more prevalent than over-dosing (Guenoun et al., 2023; Guo et al., 2023; Ruiz et al., 2018; Zhao et al., 2024; Steinberg et al., 2018). Inappropriate dosing can have adverse clinical consequences, including thromboembolism, bleeding, hospitalization, and death (Camm et al., 2020; Guo et al., 2023; Pereira et al., 2022; Chan et al., 2020). Presently, an unmet need persists for the identification of the specific determinants for full- and low-dosing in NOAC therapy, and previous studies have concentrated only on determinants for inappropriate NOAC prescription in general (Guenoun et al., 2023; Guo et al., 2023; Ruiz et al., 2018; Zhao et al., 2024; Steinberg et al., 2018). Therefore, further research is needed to construct an effective model for predicting and managing NOAC use to promote more personalized clinical decision-making.

With the extensive application of artificial intelligence (AI), the machine learning (ML) method has been increasingly applied in the medical field. Many ML techniques have been used to develop prediction models, of which most have exhibited good clinical value (Hu et al., 2024; Ding et al., 2023). Least absolute shrinkage and selection operator (LASSO) regression is a ML technique that employs L1 regularization for variable selection. By constituting a penalty function, L1 regularization forces the sum of the absolute values of the coefficients to be less than some fixed value while compressing certain coefficients to zero, thus obtaining a more refined model (Ding et al., 2023; Pavlou et al., 2016). LASSO regression has outperformed conventional methods in performance, and it can effectively select variables, reduce model complexity, and prevent over-fitting (Ding et al., 2023). However, in the area of anticoagulation therapy based on NOACs, correlational research is notably limited.

In the present study, patient outcomes were analyzed according to edoxaban dose categories, which included full-dose low-dose edoxaban therapy. We hypothesized that low-dose edoxaban use may be associated with high-risk patient backgrounds, such as demographic factors, comorbidities, laboratory characteristics, or concomitant medication use. In the present study, we constructed a LASSO regression model that includes patients’ demographic and clinical variables for the prediction of the probability of low-dose edoxaban use among hospitalized patients. As no similar study could be found in the previous literature, the present study may contribute to efforts to explore regional NOAC optimal management strategies. In addition, information regarding dose determinants for edoxaban administration represents important new knowledge, considering that this NOAC was not yet available on the market in Mainland China until 2018 (Zhao et al., 2024).

## 2 Methods

### 2.1 Study design, data sources, and patient involvement

**FIGURE 1:**
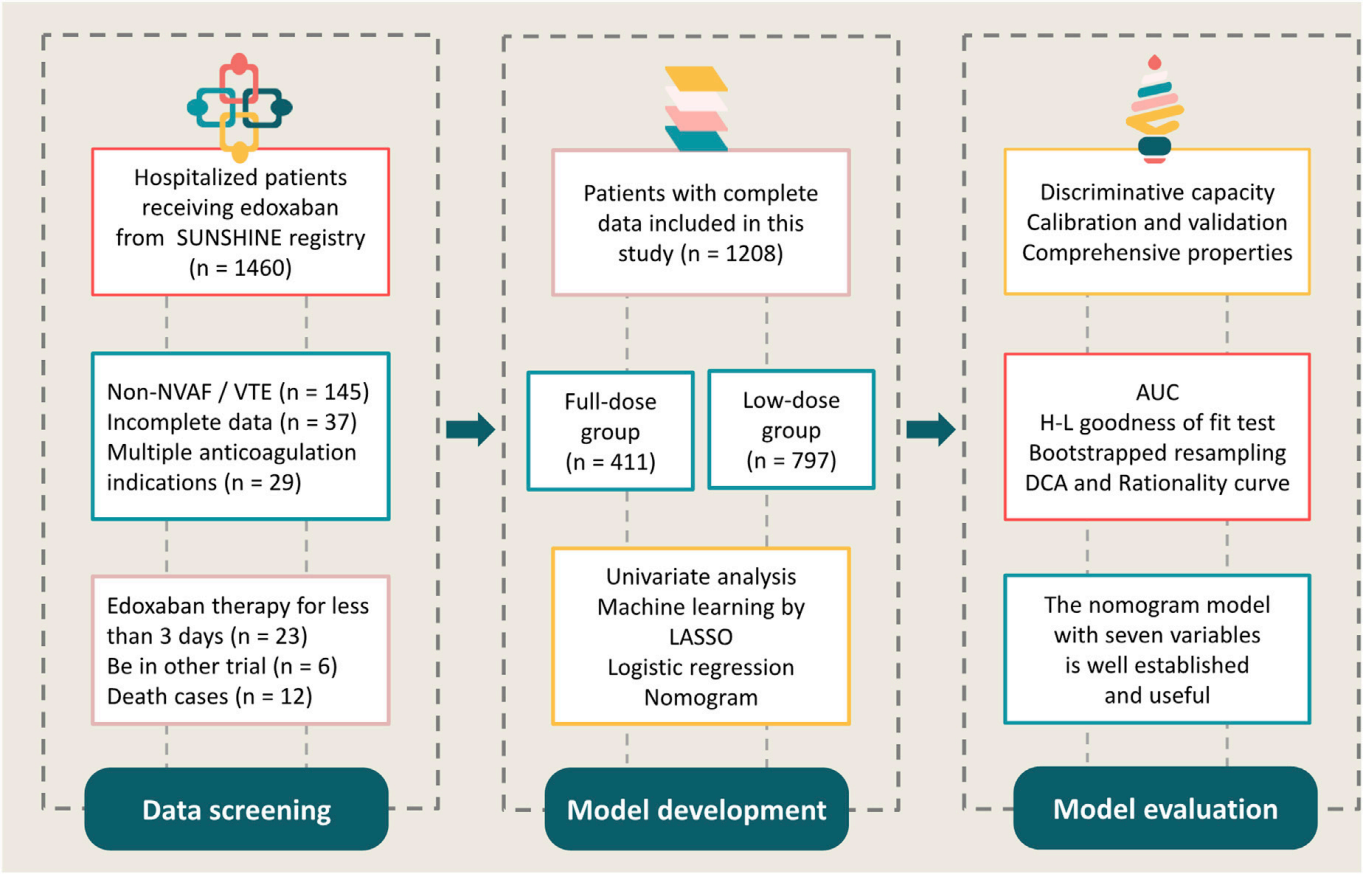
Flowchart of the processing step.

We examined the electronic medical records of hospitalized patients who were prescribed edoxaban between 1 January 2022 and 31 December 2023 in six tertiary centers from the SUNSHINE (outcomes regiStry for non-vitamin K antagonist oral anticoagUlants treatmeNt in variouS tHrombotIc dIseases for better cliNical practicE) registry, a national registration focusing on atrial fibrillation (AF), VTE, NOAC use, and relevant clinical outcomes. Additional detailed information from the SUNSHINE registry has been published previously (Zhao et al., 2024). The present study included patients who received edoxaban therapy for at least 3 days and had complete hospital records. A total of 1208 patients were included in the final analyses (Figure 1).

The study was approved by the Henan Provincial People’s Hospital Ethical Review Board with approval number: 2022-0406 and was registered at ClinicalTrials.gov (NCT 05378308). Patient inclusion was conducted according to the principles of the Declaration of Helsinki, local regulatory policies, and the International Conference on Harmonisation Good Pharmacoepidemiological and Clinical Practice Guidelines. Personal sensitive information from medical records was deleted (e.g., name, hospitalization number, or identification card number) and collected only in aggregated form; therefore, the need for informed patient consent was waived for this study.

We defined two cohorts: full-dose (60 mg) and low-dose (30/15 mg) edoxaban use. The specific information collected included: patient demographic data, duration of hospital stay, department that provided treatment, comorbidities, medical and surgical history, relevant laboratory examination, edoxaban dose and frequency, and concomitant medications. The variables included in this study are listed in Table 1. Data related to patient characteristics and hospitalized treatment was entered via a web-based online case report form. In addition, the SUNSHINE researchers received rigorous training prior to the data collection to ensure consistency.

**TABLE 1:** Demographics and characteristics between the full-dose and low-dose groups of edoxaban prescription: the SUNSHINE Registry.

	All (*n* =1208)	Full-dose group (*n* = 411)	Low-dose group (*n* = 797)	*P*
Age [median (IQR)]	71 [62 – 79]	68 [60 – 76]	73 [64 – 80]	<0.001
< 65 years (*n*, %)	361 (29.88%)	155 (37.71%)	206 (25.85%)	–
65 – 74 years (*n*, %)	372 (30.79%)	142 (34.55%)	230 (28.86%)	–
≥75 years (*n*, %)	475 (39.32%)	114 (27.74%)	361 (45.29%)	–
Weight [median (IQR)]	65 [59 – 72]	70 [63 – 75]	62 [56 – 70]	–
≤ 60 kg (*n*, %)	388 (32.12%)	42 (10.22%)	346 (43.41%)	<0.001
> 60 kg (*n*, %)	820 (67.88%)	369 (89.78%)	451 (56.59%)	
Sex (*n*, %)				<0.001
Male	644 (53.31%)	253 (61.56%)	391 (49.06%)	–
Female	564 (46.69%)	158 (38.44%)	406 (50.94%)	–
Departments (*n*, %)				0.062
Cardiovascular department	611 (50.58%)	192 (46.72%)	419 (52.57%)	–
Other department	597 (49.42%)	219 (53.28%)	378 (47.43%)	–
Surgery or operation	423 (35.02%)	118 (28.71%)	305 (38.27%)	0.001
Comorbidities (*n*, %)				–
Hypertension	684 (56.62%)	227 (55.23%)	457 (57.34%)	0.523
Coronary heart disease	521 (43.13%)	157 (38.20%)	364 (45.67%)	0.015
Congestive heart failure	421 (34.85%)	135 (32.85%)	286 (35.88%)	0.324
Cerebrovascular disease	362 (29.97%)	114 (27.74%)	248 (31.12%)	0.251
Diabetes mellitus	326 (26.99%)	99 (24.09%)	227 (28.48%)	0.118
Hyperlipidemia	177 (14.65%)	67 (16.30%)	110 (13.80%)	0.281
Indication for anticoagulation (*n*, %)				–
SPAF	784 (64.90%)	208 (50.61%)	576 (72.27%)	<0.001
VTE	424 (35.10%)	203 (49.39%)	221 (27.73%)	
DVT	254 (21.03%)	97 (23.60%)	157 (19.70%)	–
PE	87 (7.20%)	47 (11.44%)	40 (5.02%)	–
DVT plus PE	83 (6.87%)	59 (14.36%)	24 (3.01%)	–
Combined treatments (*n*, %)				–
Antiplatelet agents	264 (21.85%)	82 (19.95%)	182 (22.84%)	0.282
Aspirin	144 (11.92%)	41 (9.98%)	103 (12.92%)	–
P2Y_12_ inhibitor	54 (4.47%)	17 (4.14%)	37 (4.64%)	–
DAPT	66 (5.46%)	24 (5.84%)	42 (5.27%)	–
AADs^a^	239 (19.78%)	37 (9.00%)	202 (25.35%)	<0.001
NSAIDs	72 (5.96%)	20 (4.87%)	52 (6.52%)	0.305
SSRIs	41 (3.39%)	10 (2.43%)	31 (3.89%)	0.247
Antiepileptic drugs	34 (2.81%)	7 (1.70%)	27 (3.39%)	0.135
Antifungal drugs	34 (2.81%)	10 (2.43%)	24 (3.01%)	0.695
Creatinine clearance^b^ (*n*, %)				0.119
> 50 mL/min	956 (79.14%)	340 (82.73%)	616 (77.29%)	–
> 30 – 50 mL/min	190 (15.73%)	52 (12.65%)	138 (17.31%)	–
≤ 15 – ≤ 30 mL/min	47 (3.89%)	13 (3.16%)	34 (4.27%)	–
< 15 mL/min/dialysis/kidney transplantation	15 (1.24%)	6 (1.46%)	9 (1.13%)	–
Child Pugh level (*n*, %)				1.000
Class A	1153 (95.45%)	392 (95.38%)	761 (95.48%)	–
Class B	55 (4.55%)	19 (4.62%)	36 (4.52%)	–
Current anemia (*n*, %)	355 (29.39%)	80 (19.46%)	275 (34.50%)	<0.001
History of bleeding (*n*, %)	82 (6.79%)	22 (5.35%)	60 (7.53%)	0.192

SUNSHINE, outcomes regiStry for non-vitamin k antagonist oral anticoagUlants treatmeNt in variouS tHrombotIc dIseases for better cliNical practicE; IQR, interquartile range; SPAF, stroke prevention for atrial fibrillation; VTE, venous thromboembolism; DVT, deep vein thrombosis; PE, pulmonary embolism; DAPT, dual antiplatelet therapy; AADs, antiarrhythmic drugs; NSAIDs, non-steroidal anti-inflammatory drugs; SSRI, selective serotonin re-uptake inhibitor.

^a^ AADs included dronedarone or amiodarone.

^b^ Calculated based on Cockroft-Gault formula.

### 2.2 Outcomes

The aims of the present study were to: 1) describe anticoagulation patterns achieved with an edoxaban prescription, including the prevalence and contemporary trend of low-dose edoxaban prescription; 2) identify potential predictors of low-dose edoxaban use; and 3) develop and validate a prediction model for low-dose edoxaban use among hospitalized patients.

### 2.3 Statistical analysis

Descriptive data are reported using numbers and proportions for categorical variables, while data for continuous variables are presented as mean ± standard deviation (SD) or median and interquartile range (IQR). Differences were explored using the Chi-square test or Fisher’s exact test for categorical variables and the Student’s t-test or Mann–Whitney U-test for continuous variables as appropriate. A two-sided *P* < 0.05 indicated statistical significance.

The LASSO technique was used to select the optimal predictive variables. The optimal tuning parameter (lambda) selection in the LASSO model used 10-fold cross-validation and 1 standard error (1 se) as the minimum criterion. Multivariate logistic regression was conducted using variables confirmed by LASSO regression. The model selection was based on the backward stepwise method, using the likelihood ratio test with the Akaike information criterion (AIC) as the selection criterion [18]. We calculated the AIC at each step, stopped when further adding or removing a variable no longer improved the AIC, and from this, we obtained the model with the lowest AIC. The model with the lowest AIC was selected as the final model. Regression coefficients with standard error and odds ratio (OR) with 95% confidence intervals (CI) were calculated for each variable included in the final model as effect estimates. Based on the results of logistic regression analysis, a nomogram was constructed to provide a visual point system for estimating the probability of low-dose edoxaban use.

The area under the receiver operating characteristic (ROC) curve (AUC) was used to evaluate the discriminative performance of the model, and the Hosmer–Lemeshow (H-L) goodness of fit test was used to evaluate the model’s fit. For internal validation of the model, the bootstrap resampling method (1000 bootstrapped resampling) was used. Decision curve analysis (DCA) was applied to evaluate the clinical practicability of the model by assessing the net benefits at different threshold probabilities. We also conducted rationality curve analysis, comparing the ROC curve of the model with that of the model utilizing single predictor. Data analyses were performed using SPSS version 27.0 (IBM Corp., Armonk, NY, United States) and R software version 4.2.1 (R Foundation for Statistical Computing).

## 3 Results

### 3.1 Characteristics of hospitalized patients treated with edoxaban

The baseline characteristics, including general demographic features, comorbidities, concomitant medications, and relevant laboratory data, of the 1208 patients included in this study are presented in Table 1. The median patient age was 71 years (IQR 62–79 years), and 53.31% of the patients were male. The most common comorbidities were hypertension (56.62%) and coronary heart disease (43.13%), and 35.02% of the patients had undergone surgery or operation. Moderate impaired renal function (creatinine clearance ≤50 mL/min) was present in 20.86% of patients, and 4.55% were at the stage of hepatic dysfunction (Child Pugh’s Class B). With regard to concomitant medications, 264 patients (21.85%) were receiving antiplatelet agents, and 239 patients (19.78%) were receiving antiarrhythmic drugs (AADs), such as dronedarone or amiodarone.

Of the 1208 included patients, 411 patients (34.02%) were treated with 60 mg edoxaban therapy (full-dose group) and 797 patients (65.98%) were treated with 30 mg (n = 784, 98.37%) or 15 mg (n = 13, 1.63%) edoxaban (low-dose group). Comparison of the full- and low-dose groups showed that patients in the low-dose group were older (*P* < 0.001), weighed less (*P* < 0.001), were more likely to be female (50.94%, *P* < 0.001), and had a higher incidence of comorbidities, particularly coronary artery disease (*P* = 0.015) and anemia (*P* < 0.001). Further, differences were also observed between the full-dose group and the low-dose group in the percentages of patients treated with edoxaban as a treatment for AF (*P* < 0.001), treated by surgery or operation (*P* < 0.001), prescribed AADs (*P* < 0.001), and with a history of bleeding (*P* < 0.001).

### 3.2 Variables included in the prediction model for low-dose edoxaban treatment

**FIGURE 2:**
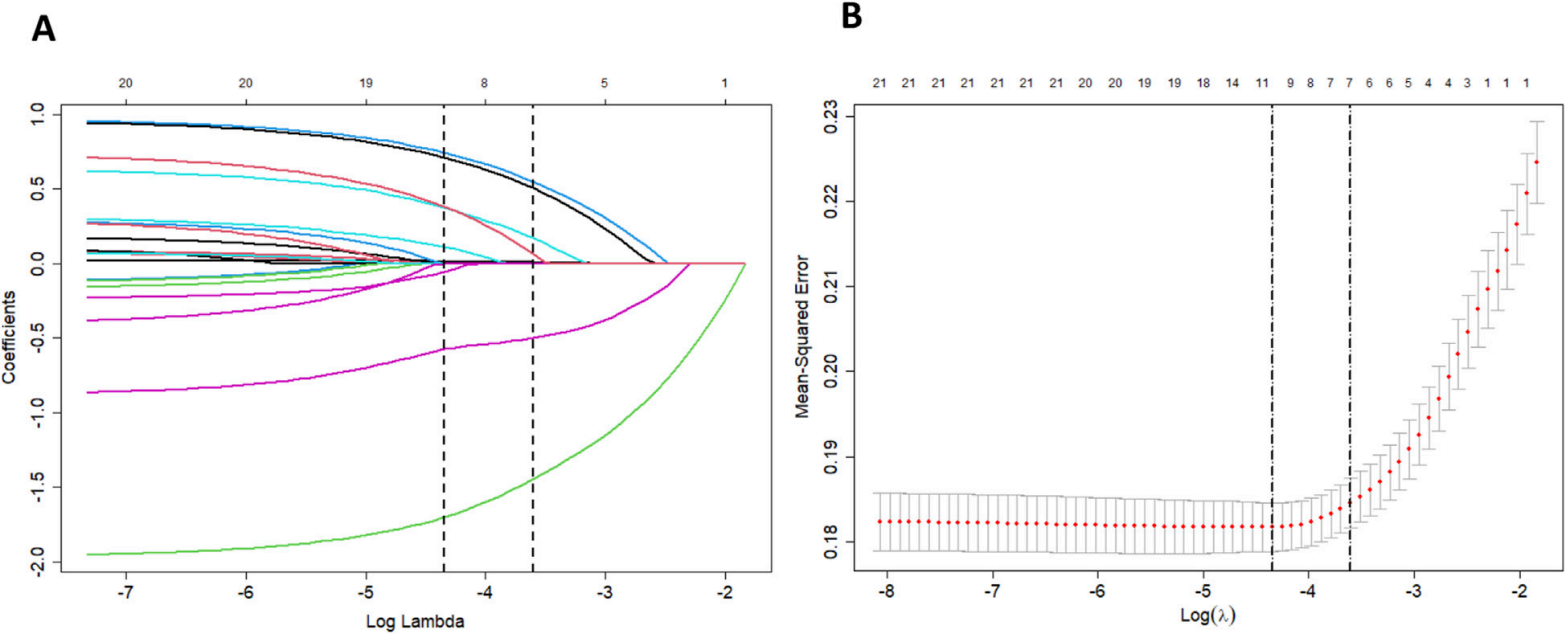
**(A)** LASSO coefficient profiles of clinical features. A coefficient profile plot was produced against the log lambda sequence. **(B)** The optimal penalization coefficient lambda was generated in the LASSO via 10-fold cross-validation. The lambda value of the 1-fold mean square error was given.

Variable selection and model complexity adjustment were performed using the LASSO regression method (Figure 2 and Table 2), and 10-fold cross-validation was conducted to select the most relevant variables based on lambda 1 se. Through this process, seven predictive variables, including age, weight, surgery or operation, anticoagulation indication, AADs, anemia, and bleeding history, were selected as the best subset of factors for use in a prediction model for low-dose edoxaban treatment.

**TABLE 2:** Predictors selected by LASSO with 10-fold cross validation.

Number of predictors	Lambda values	Predictors selected
7	Lambda (1se)	Age, weight, surgery or operation, indication for anticoagulation, AADs, current anemia, history of bleeding
12	Lambda (min)	Age, weight, department, surgery or operation, hyperlipidemia, coronary heart disease, diabetes mellitus, indication for anticoagulation, AADs, Child Pugh level, current anemia, history of bleeding

LASSO, least absolute shrinkage and selection operator; AADs, antiarrhythmic drugs.

### 3.3 Nomogram for predicting low-dose edoxaban treatment

**FIGURE 3:**
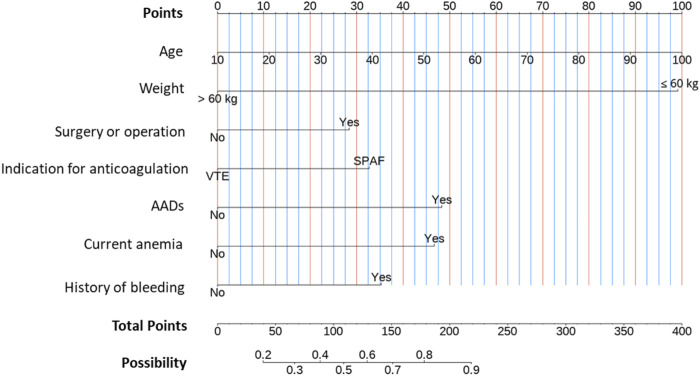
Nomogram for predicting edoxaban low-dose use.

Based on the results of the LASSO regression, a multivariate logistic regression model using the backward stepwise selection process with the AIC was developed to construct the predictive model (Table 3 and Figure 3), which was visualized as a nomogram. For each variable, a specific score is obtained on the nomogram, and the scores of individual variables are summarized to obtain the total score. The total score is translated into the possibility of low-dose edoxaban treatment by drawing a vertical line downward at the location of the total score. According to the standardized regression coefficients, age was the best predictor, followed by weight, AAD therapy, current anemia, bleeding history, anticoagulation indication, and surgery or operation (Figure 3). For example, for a 70-year-old patient (66 points for age) who had anemia (47 points) and received edoxaban and amiodarone (48 points) for AF treatment (33 points), the total score would be 194 points, with the approximate risk of 85% for low-dose edoxaban use by the nomogram model.

**TABLE 3:** Multivariate logistic regression analysis of predictors for low-dosing of edoxaban based on LASSO regression procedure.

Predictors	Beta coefficient	SE	OR (95 % CI)	*P*
Age	0.023	0.00589	1.022 (1.010-1.034)	< 0.001
Weight				
≤ 60 kg			1 (Reference)	
> 60 kg	-1.98	0.1876	0.138 (0.094-0.197)	<0.001
Surgery or operation				
No			1 (Reference)	
Yes	0.567	0.1556	1.763 (1.302-2.398)	<0.001
Indication for anticoagulation				
SPAF			1 (Reference)	
VTE	-0.655	0.1526	0.519 (0.384-0.699)	<0.001
AADs				
No			1 (Reference)	
Yes	0.962	0.2135	2.615 (1.736-4.016)	<0.001
Current anemia				
No			1 (Reference)	
Yes	0.931	0.162	2.535 (1.853-3.499)	<0.001
History of bleeding				
No			1 (Reference)	
Yes	0.63	0.2820	1.878 (1.093-3.317)	0.025

LASSO, least absolute shrinkage and selection operator; SE, standard error; OR, odds ratio; CI, confidence interval; SPAF, stroke prevention for atrial fibrillation; VTE, venous thromboembolism; AADs, antiarrhythmic drugs.

### 3.4 Evaluation of the predictive model

**FIGURE 4:**
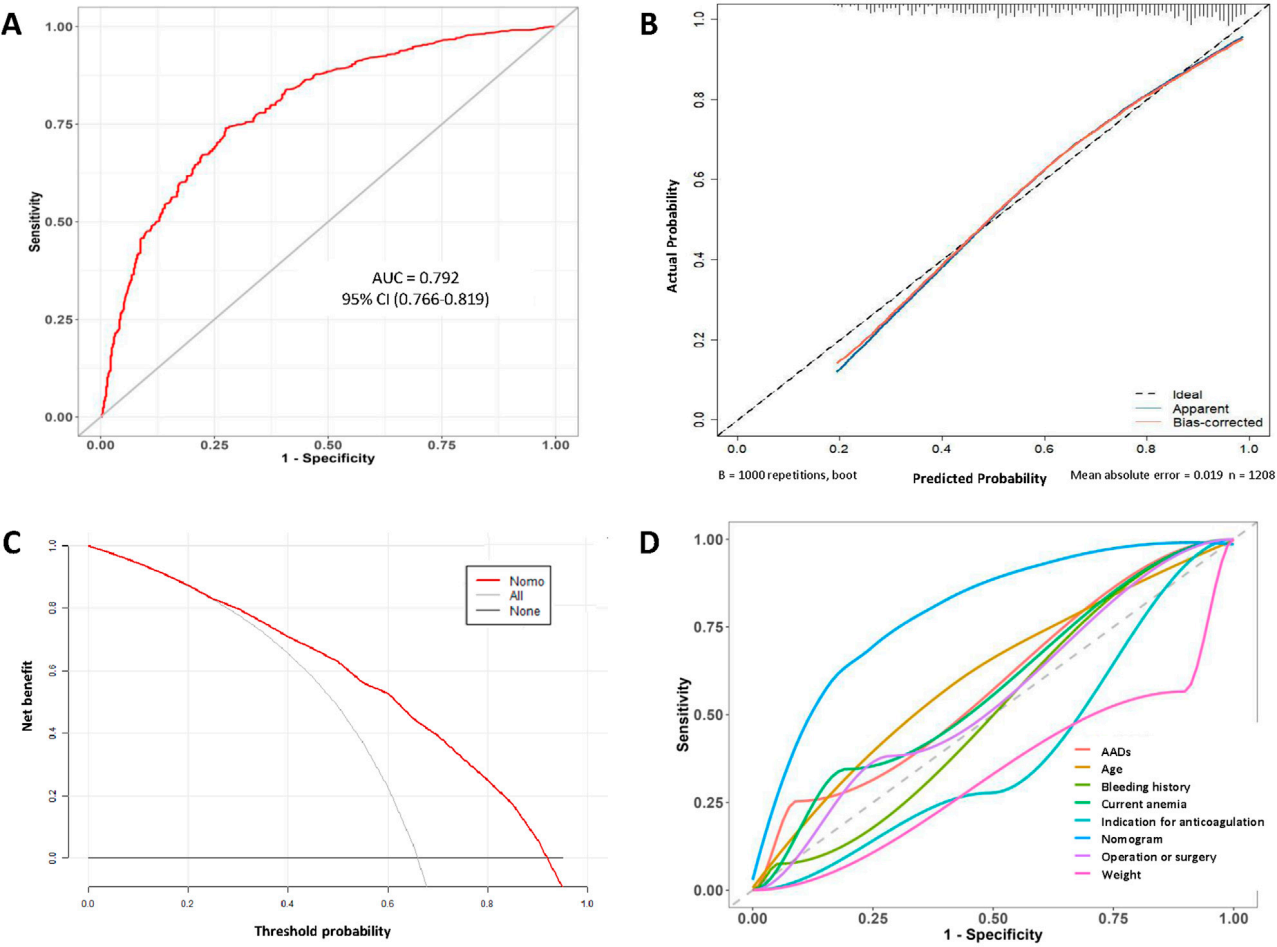
**(A)** ROC curve of the nomogram. **(B)** Calibration curve of the nomogram. **(C)** Decision curve analysis curve of the nomogram. **(D)** Rationality curve analysis of the nomogram.

The performance of the model was assessed by ROC curve analysis (Figure 4A), which showed an AUC of 0.792 (95% CI, 0.766–0.819), indicating good discriminatory ability. To reduce the optimism of the model, internal validation by the bootstrap resampling method (1000 bootstrap resamples) was conducted, and the predicted values were in good agreement with the observed values (Figure 4B). In the figure, the x-axis represents the predicted low-dose risk; the y-axis represents the actual low-dose occurrence; the diagonal dotted line represents a perfect prediction by an ideal model; and the red line represents the performance of the nomogram, for which a close fit to the ideal diagonal line indicates good predictive ability. The Hosmer–Lemeshow goodness of fit test showed that the model was well calibrated (χ^2^ = 10.757, *P* = 0.2158).

Subsequently, on DCA, the horizontal and vertical axes represent the threshold probability and net benefit, respectively. The lines of the horizontal axis and vertical axis represent the benefit of different predictive variables. The DCA curves showed a more significant net benefit than a treat all (slope line) or none (horizontal line) plan within a risk threshold of range from 0.22 to 0.95 (Figure 4C). Furthermore, we compared the ROC curve for the model with those for individual variables (Figure 4D). Significantly, the AUC values for all individual variables were consistently less than that of the model, confirming the robustness of the model. Overall, the nomogram constructed in this study performed well and showed favorable potential for clinical decision-making regarding the use of low-dose edoxaban therapy in hospitalized patients.

## 4 Discussion

Patients with NVAF or VTE are at increased risk of thrombosis due to blood stasis, endothelial injury, or hypercoagulability, which can lead to complications such as stroke or pulmonary embolism. Therefore, anticoagulation treatment with NOACs is warranted to prevent these potentially life-threatening events (Collet et al., 2021; Joglar et al., 2024; Stevens et al., 2021; Ortel et al., 2020). Few real-world studies have provided on edoxaban use among hospitalized patients to date (Zhao et al., 2024; Guenoun et al., 2023), and thus, more research was needed for this newly approved NOAC in China. To our knowledge, this is the first large-scale, observational, multicenter study to systematically investigate the incidence for low-dose edoxaban treatment and to identify the relevant risk factors among prospectively collected patients diagnosed as AF or VTE. The main findings of the present study are as follows:

First, despite the likely possibility that clinicians would be conservative when dosing or administering new therapies, we found that low-dose edoxaban use was prevalent, even among patients with AF or VTE. In this study, approximately two-thirds of the patients received reduced doses in their edoxaban regimen (65.98%). When compared with previous NOAC-relevant studies (Akao et al., 2023; Chan et al., 2019; Lee et al., 2019), a higher percentage of Chinese patients in our study were given low-dose edoxaban. The administration of edoxaban should be performed only by specific clinicians with expertise in anticoagulation management and thrombotic disorders, because its prescribing requires an in-depth understanding of patient-specific factors such as renal function, body weight, potential drug–drug interactions, and bleeding risks. This finding is therefore worthy of more attention. This discrepancy could be explained by the regional characteristics, unit culture, clinicians’ experience, the variability of practice among hospitals, and individual differences among patients. In addition, bleeding remains a major concern for clinicians (Chan et al., 2020; Chao et al., 2023). In clinical practice, especially in Asia, clinicians tend to prescribe low doses of NOACs often due to concerns over potential bleeding risk, while NOAC-reversal agents remain in limited supply (Zhao et al., 2020a).

For edoxaban, the approved dose reduction criteria consider body weight, renal function, or concomitant use of particular p-glycoprotein inhibitors (dronedarone, ciclosporin, erythromycin, or ketoconazole) (Zhao et al., 2022). A lower 15 mg dose was approved in China according on the outcome of the ELDERCARE-AF (Edoxaban Low-Dose for Elder Care-Atrial Fibrillation Patients) clinical trial for AF patients (≥80 years), who were considered to be unsuitable candidates for oral anticoagulant therapy at doses approved for stroke prevention, including those with lower body weight (≤45 kg), low creatinine clearance (15–30 mL/min), history of bleeding from a critical area or organ or gastrointestinal bleeding, continuous use of non-steroidal anti-inflammatory drugs (NSAIDs), or current use of an antiplatelet drug (Okumura et al., 2020). In contrast, edoxaban at the dose of 15 mg is not approved in Europe, the US, or other Western countries, where patients with similar clinical characteristics are typically managed with alternative strategies or by adjusting approved doses. In the present study, factors associated with the prescription of the low-dose were age, weight, surgery/operation, anticoagulation indication, AADs, current anemia, and bleeding history, and we found that clinicians did not fully follow the recommended reduction regimen on the label. These findings were similar to those of previous studies in which NOACs were withheld or given at reduced doses for patients with advanced age, frailty, bleeding risk, and multiple comorbidities (Shen et al., 2021). It is conceivable that these factors may have a strong impact on the prognosis of thrombotic diseases, but further research is needed.

Several studies have reported an association between low-dosing of NOAC and patient outcomes (Camm et al., 2020; Ciou et al., 2024; Shen et al., 2023). In an observational study (Ciou et al., 2024), among patients with increased bleeding risk but relatively low thromboembolism risk, the low-dose NOAC regimen increased the risk of ischemic stroke without reducing the major bleeding risk and showed a worse net clinical benefit than the standard-dose regimen. In another registry (Camm et al., 2020), NOAC administration at non-recommended doses was associated with increased death risk (mostly cardiovascular death), compared with recommended doses. These results suggest the need for additional efforts to improve the quality of NOAC use and effective dosing (Rymer et al., 2023).

Furthermore, in this study, we used a ML algorithm (LASSO regression) to identify a set of predictive risk factors and develop a prediction model for the use of low-dose edoxaban in hospitalized patients with AF or VTE. The risk factors identified included age, weight, surgery or operation, anticoagulation indication, AAD use, current anemia, and bleeding. The inclusion of surgery or operation as a variable is relatively novel. Notably, all parameters are readily available in routine clinical practice. Considering the large number of patients in the low-dose population, great benefit is expected from implementation of this model for risk screening.

To date, numerous studies have focused on influencing factors for inappropriate NOAC prescriptions (Moudallel et al., 2022; Rymer et al., 2023; Sanghai et al., 2020; Caso et al., 2023). Due to the lack of data or information on factors in the model, the number of variables that should be included in the model remained unclear. Although inclusion of more variables can supply more information for the model, a large number of variables may limit the clinical applicability of the model, and even some non-causal variables may decrease the accuracy of the model (Hu et al., 2024). Considering the irregular nature of the variables, the LASSO method was adopted for variable screening. More than 20 candidate predictors were evaluated, and seven independent factors were ultimately identified. The final model, established from these seven variables, may provide a valuable tool in support of clinical decision-making for hospitalized patients potentially in need of edoxaban.

Our nomogram represents the first model tailored for predicting low-dose edoxaban use in patients with AF or VTE. Early identification of risk factors for reduced dosing is crucial for timely implementation of medical interventions. Additionally, patients may be able to achieve a better prognosis and lower mortality risk with timely identification of the risk of inappropriate low-dosing administration and treatment with appropriate anticoagulation therapy. Consequently, a nomogram established based on readily available factors during hospitalization can provide valuable guidance for early administration of correct anticoagulant intervention.

The prediction model showed good performance in terms of discrimination, calibration, and clinical applicability, with an AUC value of 0.792 (95% CI 0.766–0.819). The internal evaluation using the 1000 bootstrap resampling method confirmed the accuracy of the model. Moreover, the majority of the threshold probabilities in the DCA curve demonstrated favorable net benefits, indicating the practicality of the model for identifying and managing such patients. Plausibility analysis again demonstrated the reasonableness of the model. Finally, we note that this relatively personalized model-based approach may enhance the appropriate use of NOACs by providing accurate predictions without any additional cost.

This study demonstrated the transformative potential of a ML algorithm in medical analysis. By utilizing the LASSO technique and multivariate data analysis, risk screening as well as timely medical intervention can be carried out, ultimately bringing better health outcomes for patients with AF or VTE. These findings demonstrate a crucial role for model prediction in guiding clinical decision-making, emphasizing the importance of utilizing ML techniques to promote standardized anticoagulation treatment management and the overall health improvement of patients.

## 5 Limitations

This study has several limitations. First, the robustness of the model may be affected by the lack of external validation. However, internal evaluation and validation were conducted using the bootstrap resampling method, which is an advantage of our study. Second, certain patient information has not been reported, such as the specific types of surgeries performed, additional patient-related factors and clinical information. While these omissions may restrict our ability to draw more detailed conclusions, our findings underscore the need for future studies to address these gaps and provide more granular insights. Finally, the model was developed based on data from Chinese patients, and its generalizability to other patient populations or countries is not clear.

## 6 Conclusion

We developed a predictive model based on age, weight, surgery or operation, anticoagulation indication, AAD use, anemia, and bleeding history to help evaluate the risk of low-dose edoxaban use in hospitalized patients. The model not only showed high predictive performance but also represents a new platform for personalized analysis of patients’ risk factors. Based on the evaluation results, precise interventions targeting the variable risk factors can be conducted for focus groups, with good practice-guiding implications.

## Data Availability

The original contributions presented in the study are included in the article/supplementary material, further inquiries can be directed to the corresponding author.
